# Veranderingen in ervaren voedselzekerheid en eetgedrag in Nederland sinds de COVID-19-uitbraak

**DOI:** 10.1007/s12508-021-00311-0

**Published:** 2021-07-23

**Authors:** Laura A. van der Velde, Mattijs E. Numans, Jessica C. Kiefte-de Jong

**Affiliations:** afdeling Public Health en Eerstelijnsgeneeskunde, LUMC-Campus Den Haag, Den Haag, Nederland

**Keywords:** COVID-19, eetgewoonten, voeding, voedselzekerheid, eetpatroon, COVID-19, Eating patterns, Food, Food security, Eating habits

## Abstract

In dit onderzoek beschrijven we veranderingen in ervaren voedselzekerheid en eetgewoonten in Nederland sinds het begin van de COVID-19-crisis, hoe deze veranderingen werden ervaren, en welke mogelijkheden werden aangegeven voor een gezonder eetpatroon. Het onderzoek werd uitgevoerd met online vragenlijsten onder volwassenen in Nederland met een relatief lage sociaaleconomische positie (*n* = 1.033). De resultaten laten veranderingen in ervaren voedselzekerheid zien sinds het begin van de COVID-19-crisis: zowel richting een hogere als een lagere ervaren voedselzekerheid. Ongeveer een vijfde van de deelnemers gaf een veranderd eetpatroon aan sinds het begin van de COVID-19-crisis, terwijl meer dan de helft van de deelnemers met een verminderde voedselzekerheid een veranderd eetpatroon rapporteerde: zij gaven aan minder gezond en minder groente en fruit te eten. Deelnemers met een verminderde voedselzekerheid hadden vaker behoefte aan hulp bij gezonder eten. Een goedkoper voedselaanbod werd gezien als een oplossing, terwijl voedselbankgebruik weinig werd genoemd als mogelijke hulp voor een gezonder eetpatroon. De COVID-19-crisis leidt tot financiële onzekerheid bij veel mensen en ook tot veranderingen in de beschikbaarheid van voedsel – bekende risicofactoren voor een verminderde voedselzekerheid. Daarom benadrukken deze bevindingen de urgentie van maatregelen om voedselzekerheid te verhogen en gepaste hulp en ondersteuning te bieden bij een gezond eetpatroon, vooral voor mensen met een verminderde voedselzekerheid.

**Digitaal aanvullende content:**

De online versie van dit artikel (10.1007/s12508-021-00311-0) bevat aanvullend materiaal, toegankelijk voor daartoe geautoriseerde gebruikers.

## Abstract (en)

In this study we describe changes in perceived food insecurity and eating habits in the Netherlands since the start of the COVID-19-crisis, how these changes were experienced, and which options were indicated for a healthier diet. The study was conducted through online questionnaires among adults with a relatively low socioeconomic position living in the Netherlands. We included 1033 participants. The results show changes in perceived food insecurity in the Netherlands since the start of the COVID-19 crisis, with some participants experiencing lower food security and others experiencing higher food security. About one-fifth of the participants reported that their eating pattern had changed since the start of the COVID-19-crisis, while more than half of the food insecure participants reported a changed eating pattern: in particular, they reported eating less healthily and fewer fruits and vegetables. Participants experiencing food insecurity more often reported to want help for a healthier diet. A cheaper food supply was seen as a promising solution, while food bank use was rarely mentioned in this regard. The COVID-19-crisis can lead to financial insecurity and changes in food availability/accessibility, known risk factors for food insecurity. Therefore, these findings highlight the urgency of reducing food insecurity and providing appropriate support for healthy eating, particularly for people experiencing food insecurity.

## Inleiding

In maart 2020 is het coronavirus (COVID-19) door de Wereldgezondheidsorganisatie (World Health Organisation: WHO) uitgeroepen tot een wereldwijde pandemie en sindsdien heeft de COVID-19-crisis de wereld in zijn greep. Ook Nederland is hierin niet gespaard gebleven en dit heeft verregaande gevolgen door het aantal besmettingen, maar ook vanwege de maatregelen die de Nederlandse regering heeft genomen om verspreiding van het virus tegen te gaan. De COVID-19-crisis heeft zowel mondiaal als lokaal grote sociale en economische gevolgen, en beïnvloedt ook het voedselsysteem. Stagnerende voedselaanvoer, hogere voedselprijzen en armoede (onder andere door verlies van werk en inkomen) verhogen de kans op een verminderde voedselzekerheid [1].

Met de term ‘voedselzekerheid’ wordt bedoeld dat een persoon voldoende fysieke of economische toegang heeft tot het eten dat hij of zij nodig heeft [2]. Personen die een verminderde voedselzekerheid ervaren kunnen bijvoorbeeld aangeven niet genoeg geld te hebben voor een gezonde maaltijd of om financiële redenen weleens een maaltijd over te slaan. Verschillende onderzoeken laten een afname in ervaren voedselzekerheid zien sinds het begin van de COVID-19-crisis, zoals in de Verenigde Staten en het Verenigd Koninkrijk [3, 4]. Dit is zorgwekkend omdat een verminderde voedselzekerheid langetermijngevolgen heeft. Het heeft een negatieve impact op de mentale en fysieke gezondheid, en hangt samen met een hogere prevalentie van obesitas en een minder gezond eetpatroon [5–8].

Eerder onderzoek heeft laten zien dat er ook in Nederland mensen zijn met een verminderde voedselzekerheid [9, 10]. Er is echter nog geen informatie beschikbaar over de ervaren impact van de COVID-19-crisis op voedselzekerheid in de Nederlandse context. Wel is er vooral in de grote steden een trend te zien van een toenemend beroep op voedselbankgebruik sinds het begin van de COVID-19-crisis. In december 2020 was het voedselbankgebruik met 7,2% gestegen in vergelijking met 2019. De toename was het grootst in Noord-Holland en Rotterdam [11]. De urgentie van deze kwestie is het kabinet niet ontgaan en in maart en november 2020 is extra financiële ondersteuning voor de voedselbanken beschikbaar gesteld. Hoewel voedselbankgebruik een indicatie kan geven van het aantal mensen dat verminderde toegang tot voeding heeft, blijkt uit zowel internationaal als nationaal onderzoek dat voedselbankgebruik geen accurate indicator is voor een verminderde voedselzekerheid: veel mensen die een verminderde voedselzekerheid ervaren, maken om verschillende redenen geen gebruik van de voedselbank [12, 13]. Het is daarom belangrijk om voedselbankgebruik niet als indicator te gebruiken voor het in kaart brengen van de mate van voedselzekerheid in Nederland.

Een verminderde voedselzekerheid hangt samen met een minder gezond eetpatroon, hetgeen mogelijk versterkt wordt door de COVID-19-crisis. Er is tot nu toe maar beperkt onderzoek gedaan naar mogelijke verschillen in de gevolgen van de COVID-19-crisis op eetgewoonten tussen mensen die wel of geen voedselzekerheid ervaren. Ook is er geen duidelijkheid over de gewenste of benodigde hulp en ondersteuning voor een gezonder eetpatroon in de huidige omstandigheden. Ons onderzoek richt zich daarom op de volgende vragen:

- Zijn de mate van voedselzekerheid en eetgewoonten in Nederland veranderd sinds het begin van de COVID-19-crisis en zo ja, hoe worden deze veranderingen ervaren?
- Welke mogelijkheden zijn er volgens de deelnemers om in de huidige omstandigheden gezonder te kunnen eten?

## Methode

### Onderzoekspopulatie en dataverzameling

In december 2020 werden met online vragenlijsten gegevens verzameld over de ervaren impact van de COVID-19-crisis op voedselzekerheid en eetgewoonten. De online vragenlijsten werden verstuurd naar volwassenen uit het Flycatcher-panel [14], met een over-sampling van personen met een relatief lage sociaaleconomische positie (SEP). Het Flycatcher-panel is een onafhankelijk panel dat voldoet aan de ISO-kwaliteitseisen en onder andere kan worden ingezet voor landelijk representatief onderzoek en voor onderzoek onder subgroepen [14].

### Voedselzekerheid

De mate van voedselzekerheid van de deelnemers werd bepaald met de veelgebruikte en gevalideerde *6‑item USDA Household Food Security Survey Module *[15]. De originele Engelstalige vragenlijst is door Neter en collega’s vertaald naar het Nederlands via de ‘terugvertalingstechniek’ [9]. De vragenlijst bestaat uit zes items (vijf stellingen en één frequentievraag) over omstandigheden die kenmerkend zijn voor personen die vanwege financiële redenen moeite hebben om aan basisvoedingsbehoeften te voldoen, bijvoorbeeld de vraag of de deelnemer weleens minder heeft gegeten of een maaltijd heeft overgeslagen omdat er niet genoeg geld voor eten was.

Bevestigende antwoorden op deze zes items werden opgeteld en vormden zo een schaal van 0 tot 6 punten (1 punt per bevestigend antwoord), onderverdeeld in vier categorieën: hoge voedselzekerheid (0 bevestigende antwoorden), marginale voedselzekerheid (1 bevestigend antwoord), lage voedselzekerheid (2–4 bevestigende antwoorden) en zeer lage voedselzekerheid (5–6 bevestigende antwoorden). De laatste drie categorieën werden geclassificeerd als een verminderde voedselzekerheid [16].

Om veranderingen in de mate van voedselzekerheid sinds het begin van de COVID-19-crisis te bepalen zijn er twee referentieperiodes nagevraagd: het jaar vóór de COVID-19-crisis (van maart 2019 tot maart 2020), en de periode sinds het begin van de COVID-19-crisis (vanaf maart 2020). De volledige Nederlandse vertaling van de vragenlijst over voedselonzekerheid is weergegeven in de aanvullende tab. 1 (digitaal aanvullende content). Verder is met een open vraag een eventuele toelichting gevraagd op de ervaren impact van de COVID-19-crisis op de situatie van de deelnemer wat betreft geld en voeding.

### Eetgewoonten en voedinginname

Om veranderingen in eetgewoonten sinds het begin van de COVID-19-crisis te achterhalen, gaven deelnemers op 7‑puntslikertschalen aan of zij gezonder/minder gezond; minder/meer; minder vaak/vaker op een dag; en minder of meer groente/fruit, snacks/snoep en fastfood waren gaan eten sinds de COVID-19-crisis. Daarnaast gingen we met de vragenlijst na of het kookgedrag en bestel-/afhaalgedrag van de deelnemers sinds het begin van de COVID-19-crisis was veranderd en verkregen we via open vragen een toelichting op eventuele veranderingen in het eetpatroon. De voedinginname is in kaart gebracht met een verkorte voedingsfrequentievragenlijst waarin de inname van groenten, fruit, peulvruchten, ongezouten noten, vis, graanproducten, zuivel, thee, koffie, oliën en vetten, suikerhoudende dranken, hartige snacks en zoete snacks werden nagevraagd. Hieruit is een score berekend van 0 tot 10, die aangeeft in hoeverre de inname overeenkomt met de huidige voedingsrichtlijnen [17]. Een hogere score geeft een betere overeenkomt met de richtlijnen weer. Een volledig overzicht van de voedingscomponenten met bijbehorende voedingsrichtlijnen en scoreberekening per component is weergegeven in aanvullende tab. 2 (digitaal aanvullende content).

### Hulp voor een gezonder eetpatroon

We vroegen de deelnemers aan te geven welke mogelijkheden het meest zouden helpen om een gezonder eetpatroon te bereiken. Daarbij konden ze kiezen uit een aantal opties of zelf een optie invullen. Verder hebben we de deelnemers via een open vraag de mogelijkheid geboden de gegeven antwoorden toe te lichten.

### Statistische analyse

Beschrijvende statistiek is toegepast om de mate van voedselzekerheid en de veranderingen hierin sinds het begin van de COVID-19-crisis weer te geven. Verder zijn populatiekenmerken, eetgewoonten en voedinginname beschreven, en veranderingen hierin sinds het begin van de COVID-19-crisis, zowel voor de totale populatie als voor deelnemers met een hoge of verminderde voedselzekerheid. Statistische significantie van het verschil in de status van voedselzekerheid vóór en sinds het begin van de COVID-19-crisis (continue score) is getoetst met een gepaarde t‑toets. Statistische significantie van de verschillen in eetgewoonten en voedinginname tussen deelnemers met een hoge of verminderde voedselzekerheid is getoetst met een onafhankelijke t‑toets. *P*‑waarden van < 0,05 werden beschouwd als statistisch significant. Alle resultaten zijn gebaseerd op cross-sectionele data, zodat er geen uitspraken kunnen worden gedaan over causaliteit, omdat bij dit type data niet kan worden vastgesteld of de oorzaak daadwerkelijk vooraf is gegaan aan het gevolg.

Alle analyses werden uitgevoerd met het statistische analyseprogramma IBM SPSS Statistics, versie 25.

## Resultaten

### Populatiekenmerken en de mate van voedselzekerheid

**Figure 1:**
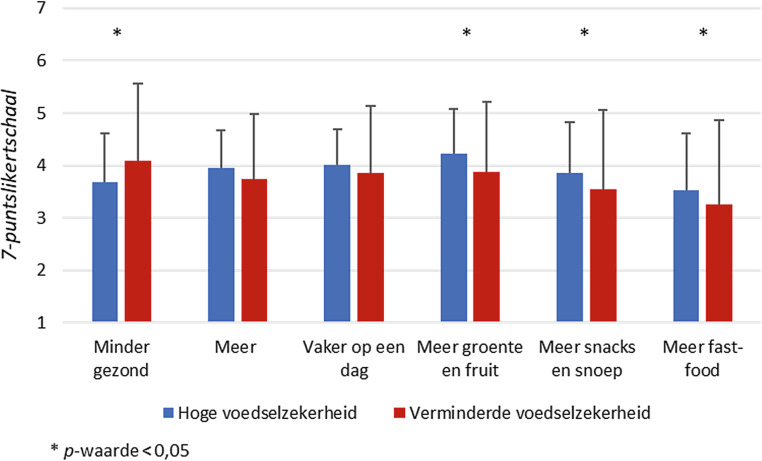

De onderzoekspopulatie bestond uit 1.033 personen. Van deze deelnemers rapporteerde 11,5% een verminderde voedselzekerheid vóór de COVID-19-crisis en 13,8% een verminderde voedselzekerheid sinds de uitbraak van COVID-19 (aanvullende fig. 1, digitaal aanvullende content). De status van voedselzekerheid vóór de COVID-19-crisis verschilde significant van de status van voedselzekerheid sinds het begin van de COVID-19-crisis (*p* < 0,001). Voor de meeste deelnemers die vóór de COVID-19-crisis een hoge of juist zeer lage voedselzekerheid ervaarden, was deze sinds het begin van de crisis niet veranderd (respectievelijk 95% en 78%, tab. 1). Voor deelnemers met een marginale en lage voedselzekerheid vonden we dat ongeveer 20% (respectievelijk 25% en 19%) een lagere voedselzekerheid rapporteerde sinds de COVID-19-crisis. Het wegvallen van werk en/of inkomen en dat (vooral gezonde) voedingsproducten sinds het begin van de COVID-19-crisis duurder zijn geworden werden als redenen genoemd, zoals hieronder geïllustreerd:

*‘Door geen werk meer te hebben, en geen inkomen elke maand heb ik wel eens geen eten gekocht! Dit was geen luiheid, maar gewoon niet voldoende geld beschikbaar. Mijn voeding was daardoor niet in evenwicht en ik sloeg wel eens een maaltijd over. Dus was ik afhankelijk.’*

*‘Ik heb gemerkt dat alles duurder is geworden, mede dankzij de verhoging van de btw, waardoor ik keuzen moet maken wat ik ga kopen om te eten en dat ik al rekenend door de winkel loop om niet bij de kassa te komen en er dan achter te komen dat ik te veel heb meegenomen … Dat is heel gênant namelijk.’*

**Table 1:** 

	Status van voedselzekerheid sinds het begin van COVID-19-crisis			
Status van voedselzekerheid vóór COVID-19-crisis (*n* (%)^a^)	Hoge voedselzekerheid	Marginale voedselzekerheid	Lage voedselzekerheid	Zeer lage voedselzekerheid
Hoge voedselzekerheid	867 (94,9)	21 (2,3)	21 (2,3)	5 (0,5)
Marginale voedselzekerheid	10 (20,0)	24 (48,0)	12 (24,0)	4 (0,8)
Lage voedselzekerheid	11 (26,2)	3 (7,1)	20 (47,6)	8 (19,0)
Zeer lage voedselzekerheid	2 (7,2)	1 (3,7)	3 (11,1)	21 (77,8)

^a^De percentages in deze tabel geven de rij-percentages aan: het percentage binnen de status van voedselzekerheid vóór de COVID-19-crisis

Een deel van de deelnemers gaf juist aan dat er sinds het begin van de COVID-19-crisis een verandering had plaatsgevonden van een marginale of lage voedselzekerheid naar een hoge voedselzekerheid (respectievelijk 20% en 26%). Als reden hiervoor werd genoemd dat er hulp was van familie, er meer thuis werd gegeten en er minder uitgaven waren (aan bijvoorbeeld uitjes, kleding en reizen), zoals hieronder geïllustreerd:

*‘Aangezien ik nergens meer heen kan en hiervoor ook geen reiskosten meer maak, weinig kleding koop en dergelijke, hou ik meer geld over om eten te kopen.’*

*‘Wij zijn in gaan wonen bij familie en hoeven niet alles zelf te bekostigen.’*

*‘Niet veel veranderd, ik woon weer thuis, waardoor ik mee kan eten met mijn ouders als ik zelf geen geld heb.’*

De ervaren voedselzekerheid nam op alle vlakken af, wat te zien is aan een toename in het aantal bevestigende antwoorden op alle stellingen (aanvullende tab. 3, digitaal aanvullende content). Meerdere deelnemers gaven aan dat zij sinds de aanvang van de COVID-19-crisis vaker goedkopere, minder gezonde en minder verse producten aten. Zo gaf een van de deelnemers aan dat er door prijsstijgingen soms niet genoeg geld was voor een maaltijd voor alle gezinsleden:

*‘De prijzen zijn omhoog en dat is echt heel duidelijk geworden. Het was al krap maar goed te doen, maar nu is het terug naar mijn jeugd en gewoonweg niet eten. Mijn kinderen […] eten dan elke maaltijd en doen ook wat ze kunnen, maar we redden het net niet met het eten. Ach, zolang zij maar eten hebben, ik kom er wel.’*

Ook gaven enkele deelnemers aan dat het door de maatregelen en adviezen om de verspreiding van het COVID-19-virus tegen te gaan, lastiger was geworden om goed op aanbiedingen te letten (bijvoorbeeld omdat er minder frequent boodschappen werden gedaan of nog maar één supermarkt werd bezocht) en dat door het ‘hamsteren’ in de supermarkten soms juist de goedkopere producten niet meer te verkrijgen waren, zoals hieronder geïllustreerd:

*‘Sinds de corona-uitbraak zijn a: de boodschappen weer veel duurder geworden. Waardoor het moeilijker rondkomen is. En dankzij de periodes waarin mensen massaal gingen hamsteren hebben wij wel eens zonder boodschappen gezeten. Omdat de normaal wat goedkopere producten gewoon uitverkocht waren. We moesten dan vaak de duurdere of andere producten halen, waar we het budget dus niet voor hebben.’*

De man-vrouwverdeling was ongeveer gelijk in de totale onderzoekspopulatie (53% man), terwijl relatief veel vrouwen een verminderde voedselzekerheid ervaarden (70%, tab. 2). Vergeleken met deelnemers met een hoge voedselzekerheid hadden deelnemers met een verminderde voedselzekerheid vaker een leeftijd van vijftig jaar of jonger en waren ze vaker alleenstaand. Voor de meeste deelnemers was het lichaamsgewicht niet veranderd sinds het begin van de COVID-19-crisis (66%). Het aantal deelnemers met een normaal gewicht was ongeveer gelijk tussen deelnemers met een hoge en verminderde voedselzekerheid, maar deelnemers met een verminderde voedselzekerheid werden wel vaker als obees geclassificeerd (dat wil zeggen een BMI > 30) (36% versus 23%). Daarnaast rapporteerden deelnemers met een verminderde voedselzekerheid vaker te zijn afgevallen (23% versus 13%) en vooral vaker te zijn aangekomen (37% versus 16%) sinds het begin van de COVID-19-crisis. Deelnemers met een verminderde voedselzekerheid rapporteerden ook vaker een verminderd inkomen (26% versus 6%) en verlies van baan (11% versus 3%) sinds het begin van de COVID-19-crisis. Ook rookten deelnemers met een verminderde voedselzekerheid aanzienlijk vaker en waren ze sinds het begin van de COVID-19-crisis vaker begonnen met roken. Een klein deel van de deelnemers gaf aan gebruik te maken van de voedselbank of hier in het verleden gebruik van te hebben gemaakt (4%, tab. 2).

**Table 2:** 

	Totale populatie	Hoge voedselzekerheid	Verminderde voedselzekerheid
Geslacht (*n* (%) man)	542 (52,5)	499 (56,1)	43 (30,1)
*Leeftijd*			
< 50 jaar	356 (34,5)	269 (30,2)	87 (60,8)
50–65 jaar	322 (31,2)	283 (31,8)	39 (27,3)
≥ 65 jaar	355 (34,4)	338 (38,0)	17 (11,9)
*Geboorteland (n (%) Nederland)*	999 (96,7)	869 (97,6)	130 (90,9)
*Burgerlijke staat*			
Gehuwd/samenwonend met kinderen	202 (19,6)	174 (19,4)	28 (19,6)
Gehuwd/samenwonend zonder kinderen	408 (39,5)	376 (42,2)	32 (22,4)
Alleenstaand met kinderen	101 (9,8)	68 (7,6)	33 (23,1)
Alleenstaand zonder kinderen	285 (27,6)	240 (27,0)	45 (31,5)
Anders	37 (3,6)	32 (3,6)	5 (3,5)
*Opleidingsniveau*			
Laag	469 (45,4)	414 (46,5)	55 (38,5)
Middel	506 (49,0)	423 (47,5)	83 (58,0)
Hoog	58 (5,6)	53 (6,0)	5 (3,5)
*Verandering baan sinds corona-uitbraak*			
Baan kwijtgeraakt	37 (3,6)	22 (2,5)	15 (10,5)
Baan gekregen	22 (2,1)	17 (1,9)	5 (3,5)
Gelijk gebleven	974 (94,3)	851 (95,6)	123 (86,0)
*Verandering inkomen sinds corona-uitbraak*			
Minder inkomen	88 (8,5)	51 (5,7)	37 (25,9)
Meer inkomen	27 (2,6)	24 (2,7)	3 (2,1)
Gelijk gebleven	888 (86,0)	791 (88,9)	97 (67,8)
Weet niet/wil niet zeggen	30 (2,9)	24 (2,7)	6 (4,2)
*Verandering schuldhulpverleningstraject sinds corona-uitbraak*			
Ja, sinds corona-uitbraak	3 (15,8)	1 (12,5)	2 (18,2)
Nee, volgde het al voor de corona-uitbraak	16 (84,2)	7 (87,5)	9 (81,1)
Gelijk gebleven	974 (94,3)	851 (95,6)	123 (86,0)
*Roken*			
Ja, ik rook en rookte al vóór de corona-uitbraak	173 (16,7)	128 (14,4)	45 (31,5)
Ja, ik rook sinds de corona-uitbraak (hiervoor rookte ik niet)	10 (1,0)	5 (0,6)	5 (3,5)
Nee, ik rook niet maar rookte wel vóór de corona-uitbraak	190 (18,4)	163 (18,3)	27 (18,9)
Nee, ik heb nooit gerookt	660 (63,9)	594 (66,7)	66 (46,2)
*BMI*			
Normaal gewicht	404 (39,1)	354 (39,8)	50 (35,0)
Overgewicht	370 (35,8)	328 (36,9)	42 (29,4)
Obesitas	259 (25,1)	208 (23,4)	51 (35,7)
*Verandering lichaamsgewicht sinds corona-uitbraak*			
Afgevallen	145 (14,0)	112 (12,6)	33 (23,1)
Aangekomen	196 (19,0)	143 (16,1)	53 (37,1)
Gelijk gebleven	682 (66,0)	626 (70,3)	56 (39,2)
Weet niet/wil niet zeggen	10 (1,0)	9 (1,0)	1 (0,7)
*Voedselbank*			
Ja, ik maak gebruik van de voedselbank en deed dit ook al vóór de corona-uitbraak	7 (0,7)	3 (0,3)	4 (2,8)
Ja, ik maak gebruik van de voedselbank sinds de corona-uitbraak (hiervoor maakte ik er geen gebruik van)	2 (0,2)	1 (0,1)	1 (0,7)
Nee, ik maak geen gebruik van de voedselbank maar deed dit wel vóór de corona-uitbraak	35 (3,4)	19 (2,1)	16 (11,2)
Nee, ik heb nooit gebruikgemaakt van de voedselbank	989 (95,7)	867 (97,4)	122 (85,3)

### Veranderingen in eetgewoonten sinds het begin van de COVID-19-crisis

Deelnemers die een verminderde voedselzekerheid ervaarden, meldden vaker minder gezond te eten sinds het begin van de COVID-19-crisis, vooral minder groente en fruit. Ze gaven verder aan iets minder snacks, snoep en fastfood te eten vergeleken met voedselzekere deelnemers (fig. 1). Een deelnemer gaf bijvoorbeeld aan minder gezond te eten omdat er door het thuiswerken minder structuur in de dag zat. Een andere deelnemer gaf aan minder gezond te eten vanwege de hogere kosten van gezonde producten:

*‘Meer ongezond, zoals friet of pizza. Omdat de kosten van vlees en groenten te hoog zijn geworden.’*

Ook gaven sommige deelnemers aan goedkoper en/of minder te eten omdat er niet genoeg geld was, zoals deze deelnemers aangeven:

*‘Ik eet minder omdat ik ook minder geld heb om uit te geven.’*

*‘Eet veel minder, fruit koop ik niet meer en groenten bijna ook niet […]’*

**Figure 2:**
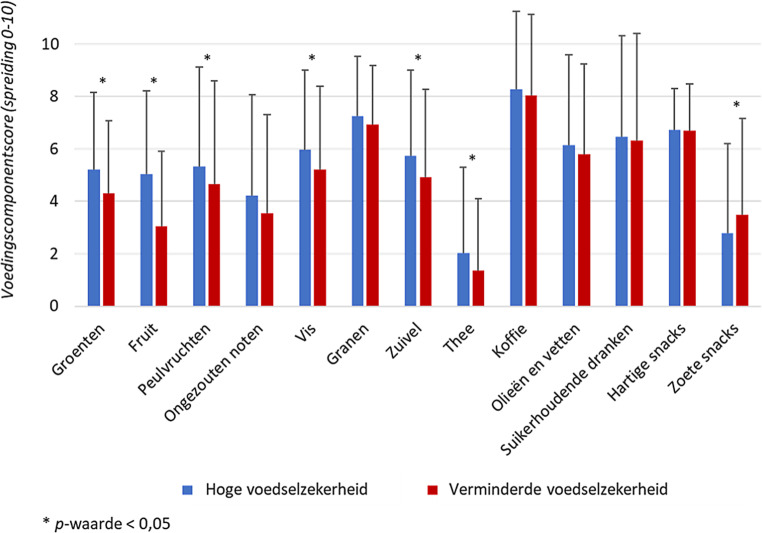

Over het algemeen rapporteerden deelnemers die een verminderde voedselzekerheid ervaarden een voedinginname die minder goed voldeed aan de voedingsrichtlijnen voor een gezond eetpatroon (fig. 2). Vergeleken met deelnemers met een hoge voedselzekerheid scoorden deelnemers met een verminderde voedselzekerheid significant lager op de componenten groenten, fruit, peulvruchten, vis, zuivel en thee, terwijl zij iets hoger scoorden op de component zoete snacks. Ook voor de overige componenten scoorden deelnemers met een verminderde voedselzekerheid over het algemeen minder goed of vrijwel gelijk aan deelnemers met een hoge voedselzekerheid.

Van de gehele onderzoekspopulatie gaf 22% aan dat het eetpatroon was veranderd sinds het begin van de COVID-19-crisis. Voor deelnemers met een verminderde voedselzekerheid was dit meer dan de helft (54%, tab. 3). De meeste deelnemers gaven aan vijf dagen per week of meer zelf te koken en voor de meeste deelnemers was dit sinds het begin van de COVID-19-crisis niet veranderd (87%). Vergeleken met voedselzekere deelnemers rapporteerden deelnemers met een verminderde voedselzekerheid vaker een verandering in kookgedrag sinds het begin van de COVID-19-crisis: zij gaven frequenter aan vaker zelf te koken (14% versus 7%) en ook gaven ze frequenter aan minder vaak zelf te koken (8% versus 4%). Verder gaven deelnemers met een verminderde voedselzekerheid vaker aan sinds het begin van de COVID-19-crisis minder vaak eten te bestellen (13% versus 5%). Wanneer er eten werd besteld, kozen deelnemers met een verminderde voedselzekerheid vaker grillgerechten en pizza/pasta, en minder vaak Thais/Chinees eten vergeleken met voedselzekere deelnemers (tab. 3).

**Table 3:** 

	Totale populatie (*N* = 1.033)	Hoge voedselzekerheid (*N* = 890)	Verminderde voedselzekerheid (*N* = 143)
*Verandering eetpatroon sinds corona-uitbraak*			
Is uw eetpatroon veranderd sinds de corona-uitbraak? (*n* (%) ja)	224 (21,7)	147 (16,5)	77 (53,8)
*Eten koken*			
Hoe vaak kookt u zelf uw hoofdmaaltijd?			
Ik kook nooit zelf mijn hoofdmaaltijd	101 (9,8)	89 (10,0)	12 (8,4)
Minder dan 1 dag per week	52 (5,0)	51 (5,7)	1 (0,7)
1–2 dagen per week	77 (7,5)	67 (7,5)	10 (7,0)
3–4 dagen per week	178 (17,2)	144 (16,2)	34 (23,8)
5–6 dagen per week	318 (30,8)	272 (30,6)	46 (32,2)
Elke dag	307 (29,7)	267 (30,0)	40 (28,0)
*Is uw kookgedrag veranderd sinds de corona-uitbraak?*			
Ja, ik kook vaker zelf	85 (8,3)	65 (7,3)	20 (14,0)
Ja, ik kook minder vaak zelf	45 (4,4)	33 (3,7)	12 (8,4)
Nee, dit is gelijk gebleven	900 (87,4)	789 (89,0)	111 (77,6)
*Eten bestellen/afhalen*			
Hoe vaak eet u afhaal-/besteld eten als hoofdmaaltijd?			
Ik haal of bestel nooit eten als hoofdmaaltijd	430 (41,6)	378 (42,5)	52 (36,4)
Minder dan 1 dag per week	504 (48,8)	432 (48,5)	72 (50,3)
1–2 dagen per week	82 (7,9)	65 (7,3)	17 (11,9)
3–4 dagen per week	13 (1,3)	13 (1,5)	0
5–6 dagen per week	2 (0,2)	1 (0,1)	1 (0,7)
Elke dag	2 (0,2)	1 (0,1)	1 (0,7)
*Is uw afhaal-/bestelgedrag veranderd sinds de corona-uitbraak?*			
Ja, ik haal of bestel vaker eten	102 (9,9)	90 (10,1)	12 (8,4)
Ja, ik haal of bestel minder vaak eten	62 (6,0)	43 (4,8)	19 (13,3)
Nee, dit is gelijk gebleven	867 (84,1)	755 (85,0)	112 (78,3)
*Wat voor soort eten bestelt u meestal/haalt u meestal af?*			
Fastfood/snackbar	126 (20,9)	107 (20,9)	19 (20,9)
Grillgerechten	72 (11,9)	58 (11,3)	14 (15,4)
Pizza/pasta	98 (16,3)	74 (14,5)	24 (26,4)
Chinees/Thais eten	194 (32,2)	176 (34,4)	18 (19,8)
Sushi/poké bowl	31 (5,1)	24 (4,7)	7 (7,7)
Salade	6 (1,0)	6 (0,7)	0
Anders	76 (12,6)	67 (13,1)	9 (9,9)

### Hulp voor een gezonder eetpatroon

**Figure 3:**
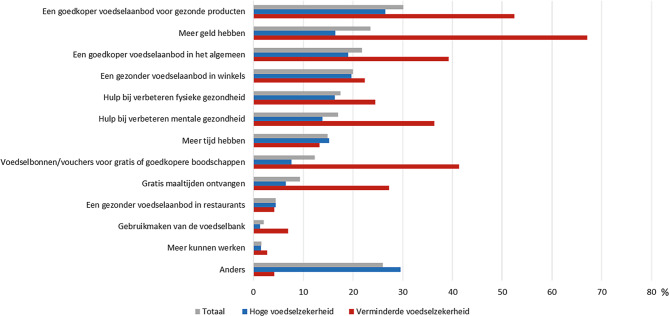

Deelnemers rapporteerden verschillende mogelijkheden die hen (of het gezin) zouden kunnen helpen om gezond(er) te eten. De meest genoemde opties waren een goedkoper voedselaanbod voor gezonde producten (30%), meer geld hebben (24%) en een goedkoper voedselaanbod in het algemeen (22%, fig. 3). Deelnemers met een verminderde voedselzekerheid hadden vaker behoefte aan vrijwel alle genoemde vormen van hulp bij gezonder eten en de door hen genoemde opties hadden veelal te maken met financiële factoren. Gebruik van de voedselbank werd door weinig deelnemers genoemd als mogelijke hulp voor een gezonder eetpatroon (1% van de deelnemers met een hoge voedselzekerheid, 7% van de deelnemers met een verminderde voedselzekerheid). Een deelnemer gaf wat dit betreft aan blij te zijn met de hulp die de voedselbank biedt, maar wel meer vet- en suikerrijke producten te consumeren vanwege het aanbod in de voedselbankpakketten:

‘*Omdat ik niet veel geld heb ben ik blij met de voedselbank, maar daar worden geen vet- of suikervrije producten gegeven en dat merk ik in mijn gewicht. Zelf kocht ik altijd 0% vet yoghurt of magere kwark en Optimel-zuiveldrank en 20+-kaas. Nu eet en drink ik alles met suiker en vet, en vaak wit brood*.’

Deelnemers noemden ook andere opties die zouden kunnen helpen bij een gezond(er) eetpatroon, zoals een groter en voordeliger aanbod van biologische voedingsmiddelen. Zoals een deelnemer aangaf:

*‘Biologische producten en gezonde voeding kosten vaak meer dan de “normale” producten. Ik kan me dat niet vaak permitteren*.*’*

Ook begeleiding van een voedingsdeskundige en een beter aanbod van kleine verpakkingen voor alleenstaanden werden genoemd. Een goedkoper en gezonder voedingsaanbod werd het meest benadrukt door de deelnemers. Zo gaf een deelnemer aan minder groente en fruit te eten vanwege de hoge kosten:

*‘Groente en fruit is héél duur. Dus is maar een voorbeeld: dan eet ik een halve banaan. Want dan heb ik twee dagen iets. Of voeg ik geen groente toe. Of vaak ook geen vlees. En eet ik dus alleen aardappels.’*

Andere deelnemers gaven aan minder gezond te eten omdat dat goedkoper is:

*‘Ongezond eten is helaas veel goedkoper. Ik moet het met mijn vijf kinderen doen van 60 euro per week, dus moet ik vaak slechte keuzen maken (waar ik niet achter sta) om de kinderen toch eten te kunnen geven.’*

*‘De gezonde dingen zijn duurder dan ongezonde dingen en dan pak je toch sneller de ongezonde dingen.’*

Verder werd een gezonder voedselaanbod in supermarkten als mogelijkheid aangegeven voor een gezondere samenleving:

*‘Als de supermarkten de overbodige zoete en zoute en bewerkte producten uit de schappen zou halen waren alle Nederlanders op slag gezonder!’*

*‘Als er meer gezonde producten in winkels komen, bijvoorbeeld zoutarme kant-en-klaarmaaltijden en vleesvervangers, enzovoort, dan zou het makkelijker zijn om dingen die je lekker vindt op een verantwoorde manier te eten*.’

## Beschouwing

De resultaten van dit vragenlijstonderzoek lieten zien dat er veranderingen werden gerapporteerd in de ervaren voedselzekerheid in Nederland in de periode vanaf het begin van de COVID-19-crisis, vergeleken met de periode vóór de COVID-19-crisis. Daarbij rapporteerde een iets hoger percentage deelnemers een verminderde vervaren voedselzekerheid sinds het begin van de COVID-19-crisis, vergeleken met de periode daarvoor. Ongeveer een vijfde van de deelnemers gaf aan dat het eetpatroon sinds de COVID-19-crisis was veranderd, terwijl meer dan de helft van de deelnemers die een verminderde voedselzekerheid ervaarden een veranderd eetpatroon rapporteerden. Zij gaven aan sinds de COVID-19-crisis vooral minder gezond en minder groente en fruit te eten. Een goedkoper en gezonder voedselaanbod werd veel genoemd als mogelijke hulp voor een gezonder eetpatroon. Deelnemers met een verminderde voedselzekerheid hadden meer behoefte aan verschillende vormen van hulp bij gezonder eten en de door hen genoemde opties voor hulp hadden vaker te maken met financiële factoren.

Een stijgend aantal rapportages en onderzoeken voorspelt bij populaties over de gehele wereld een toename of laat al een toename zien van personen die door de COVID-19-crisis een verminderde voedselzekerheid ervaren. Het hogere percentage deelnemers in ons onderzoek dat sinds het begin van de COVID-19-crisis een verminderde voedselzekerheid ervaart, vormt een bevestiging van eerder internationaal onderzoek uit onder andere de Verenigde Staten, het Verenigd Koninkrijk en Australië [3, 4, 18, 19]. Een vermindering in ervaren voedselzekerheid is zorgwekkend, omdat een verminderde voedselzekerheid schadelijke langetermijngevolgen voor de gezondheid heeft [5–8].

Uit de resultaten bleek verder dat er naast deelnemers die sinds het begin van de COVID-19-crisis een lagere voedselzekerheid ervaarden, ook een groep deelnemers was die juist een hogere voedselzekerheid ervaarde. Dat leek onder andere verklaard te worden door ondersteuning vanuit het sociale netwerk, waarbij familie bijvoorbeeld hulp bood. Ook eerder onderzoek laat zien dat sociale steun (zoals instrumentele steun door vrienden en familie) en sociale cohesie (zoals verbondenheid tussen bewoners op wijkniveau, waardoor ze elkaar steun kunnen bieden bij het verkrijgen van voedsel of het zoeken naar beschikbare maatschappelijke diensten) belangrijk zijn in het verhogen van ervaren voedselzekerheid [20]. Hieruit volgt dat het verbeteren van sociale steun en sociale cohesie mogelijk betere strategieën zijn om voedselzekerheid te verhogen, dan traditionele vormen van voedselhulp. Dit is des te belangrijker in de context van de COVID-19-crisis en de maatregelen om verspreiding van COVID-19 tegen te gaan, in het bijzonder social distancing-maatregelen, zoals het beperken van sociale bijeenkomsten en contacten. Deze maatregelen zijn belangrijk voor het tegengaan van besmettingen, maar verhogen ook het risico op sociale isolatie en eenzaamheid [21]. Pantell en Shields-Zeeman raden zorgverleners dan ook aan om bij patiënten bij wie sociale isolatie of eenzaamheid wordt waargenomen, ook rekening te houden met sociale risicofactoren, zoals voedselonzekerheid [21]. Onze resultaten bekrachtigen deze aanbeveling.

Net als eerder Nederlands onderzoek laten onze resultaten zien dat deelnemers met een verminderde voedselzekerheid vaker obesitas hadden [10], maar ook dat hun lichaamsgewicht sinds het begin van de COVID-19-crisis vaker veranderd is. Het valt op dat zij niet alleen vaker zijn afgevallen (bijvoorbeeld door minder te eten en maaltijden over te slaan), maar vooral vaker zijn aangekomen (bijvoorbeeld door een minder gezond eetpatroon). De COVID-19-crisis lijkt hiermee het al bekende verband tussen een verminderde voedselzekerheid en obesitas te versterken, en maakt het bevorderen van een gezond gewicht in deze groep nog urgenter.

Zoals eerder onderzoek liet zien hadden deelnemers die een verminderde voedselzekerheid ervaarden een minder gezond eetpatroon en in het bijzonder een lagere groente- en fruitinname [8, 13]. In lijn met recent Nederlands onderzoek gaf ongeveer 20% van de deelnemers aan dat hun eetpatroon sinds de COVID-19-crisis was veranderd [22]. Dit percentage was aanzienlijk hoger onder deelnemers die een verminderde voedselzekerheid ervaarden: hiervan gaf meer dan de helft aan dat hun eetpatroon sinds de COVID-19-crisis was veranderd. Zij rapporteerden vooral minder gezond, en minder groente en fruit te eten. Een veelgenoemde reden hiervoor was dat gezond eten, zoals groente en fruit, (te) duur werd gevonden, zoals ook naar voren kwam in eerder Nederlands onderzoek onder personen met een verminderde ervaren voedselzekerheid [23]. Zij noemden een goedkoper voedselaanbod van gezonde producten en over meer geld beschikken het vaakst als opties voor een gezonder eetpatroon. Een betaalbare en gezonde voedselomgeving was al belangrijk, maar is in deze crisis extra belangrijk vanwege het syndemische effect dat de COVID-19-crisis heeft, zowel in relatie tot andere leefstijlgerelateerde aandoeningen, als tot sociaaleconomische ongelijkheid [24].

De Nederlandse rijksoverheid speelt via beleid en wet- en regelgeving een belangrijke rol in het creëren van een betaalbare en gezonde voedselomgeving. Uit een onlangs uitgebracht onderzoeksrapport blijkt echter dat de Nederlandse rijksoverheid hierin kansen laat liggen [25]. Het rapport geeft aanbevelingen voor beleidsverbetering, waaronder het verlagen van prijzen van gezonde voedingsmiddelen, zoals groente en fruit, het verhogen van prijzen van ongezonde voedingsmiddelen, zoals suikerhoudende dranken, een groter aandeel gezonde producten in supermarkten en restaurants, en bij andere aanbieders, en het financieren van voedselhulp (zoals het verstrekken van vouchers voor het kosteloos afnemen van gezonde voedingsmiddelen aan mensen onder een bepaalde inkomensgrens) [25]. Deze aanbevelingen liggen in lijn met de in ons onderzoek gerapporteerde mogelijkheden voor hulp bij een gezonder eetpatroon. Dat wijst er ook op dat deze beleidsacties een positief effect kunnen hebben bij het verbeteren van het eetpatroon van de kwetsbare groep personen met een verminderde voedselzekerheid. Hiermee kunnen deze acties ook bijdragen aan het verkleinen van sociaaleconomische gezondheidsverschillen. Er is echter meer onderzoek nodig naar de meest effectieve vorm voor implementatie van deze beleidsacties. Een veelgenoemde oplossing voor goedkopere gezonde voeding is bijvoorbeeld het verlagen van het btw-tarief op groente en fruit. Deze maatregel is echter onuitvoerbaar verklaard door de Belastingdienst en daarnaast is het niet zeker dat een verlaging van het btw-tarief op groente en fruit van 4% (binnen de huidige Europese wetgeving is alleen een verlaging van 9% naar 5% mogelijk) daadwerkelijk effect zal hebben op de prijs die de consument betaalt en de inname van groente en fruit [26].

Een opmerkelijke uitkomst van dit onderzoek was dat maar een klein deel van de deelnemers, onder wie ook de deelnemers met een verminderde ervaren voedselzekerheid, gebruikmaakte van de voedselbank en dat voedselbankgebruik ook niet vaak werd aangemerkt als mogelijke hulp voor een gezonder eetpatroon, terwijl dit zowel nationaal als internationaal de meest bekende en toegepaste vorm is van voedselhulp [27]. Deze bevinding komt echter overeen met eerder onderzoek waaruit blijkt dat lang niet alle personen met een verminderde ervaren voedselzekerheid ook gebruik (willen) maken van de voedselbank [12, 23]. Dit kan verschillende redenen hebben. In dit onderzoek werd vooral aangegeven dat de inhoud van de aangeboden voedselbankpakketten niet altijd aan de wensen voldeed, vooral wat betreft gezonde producten. Dit kwam ook naar voren in eerdere gesprekken met personen die een verminderde voedselzekerheid ervaren [23]. Uiteraard is het voor voedselbanken een uitdaging om voeding aan te bieden met een hoge nutritionele waarde, onder andere omdat ze afhankelijk zijn van donaties [27]. Eerder onderzoek liet zien dat de producten verstrekt in voedselbankpakketten in Nederland niet voldoen aan de voedingsrichtlijnen en niet altijd voldoen aan de wensen van de gebruikers [28, 29]. Daarnaast toont systematisch literatuuronderzoek aan dat voedselbankgebruik onvoldoende helpt om voedselzekerheid te verhogen [27]. Dit geeft aan dat de huidige vorm van voedselhulp via de voedselbank niet volledig lijkt aan te sluiten op de behoeften van personen met een verminderde ervaren voedselzekerheid. Andere vormen van hulp of een aangepaste vorm van voedselhulp, zoals een sociale supermarkt (een concept waar al in verschillende steden mee wordt geëxperimenteerd, zoals in Amsterdam) sluiten mogelijk beter aan op de behoeften van personen die een verminderde voedselzekerheid ervaren [30].

Verder gaf een groot deel van de deelnemers met een verminderde voedselzekerheid aan dat het voor het handhaven van een gezond(er) eetpatroon zou helpen wanneer ze over meer geld zouden beschikken. Dat onderstreept het belang van financiële draagkracht voor deze mensen. Beschikbare inkomensvoorzieningen zijn echter juist voor mensen met een laag inkomen en/of schulden vaak te gecompliceerd, waardoor zij er niet optimaal gebruik van kunnen maken. Ook hier liggen dus kansen voor hulpverleners en beleidsmakers voor verbetering van de ondersteuning van mensen met een laag inkomen en/of schulden [31].

### Sterke punten en beperkingen

Dit onderzoek kent verschillende sterke punten. Ten eerste is dit onderzoek, voor zover ons bekend, het eerste naar veranderingen in de mate van voedselzekerheid en eetgewoonten in Nederland sinds het begin van de COVID-19-crisis. We hebben een groot aantal deelnemers kunnen includeren en de mate van voedselzekerheid is bepaald met een gevalideerd en betrouwbaar meetinstrument, dat in westerse landen als de gouden standaard wordt beschouwd [32].

Beperkingen van dit onderzoek zijn onder andere het gebruik van een panel van respondenten dat niet volledig representatief is voor Nederland. De focus van dit onderzoek lag op een populatie met een relatief lage SEP en geeft dus een relatieve overschatting van deze populatie. Ook was er zeer weinig variatie in geboorteland: het overgrote deel van de deelnemers was in Nederland geboren, terwijl eerder onderzoek laat zien dat een verminderde voedselzekerheid relatief vaker voorkomt bij etnische minderheden [33]. Daardoor kan de gevonden prevalentie een onderschatting zijn van de werkelijke mate van voedselzekerheid in deze populatie. Het grote aandeel deelnemers met Nederland als geboorteland kan verklaard worden doordat de vragenlijst in het Nederlands werd aangeboden, waardoor deze alleen kon worden ingevuld door mensen die voldoende vaardig waren in de Nederlandse taal. Daarnaast werd de vragenlijst online aangeboden, waardoor geen hulp geboden kon worden bij het invullen. De bevindingen die in dit onderzoek zijn beschreven zijn gebaseerd op zelfgerapporteerde en cross-sectionele data. Hierdoor kunnen er geen uitspraken worden gedaan over causaliteit en het kan leiden tot vertekening van de resultaten, bijvoorbeeld door sociaal wenselijke antwoorden en problemen met de herinnering. Zo kan het voorkomen dat mensen in het algemeen zich bepaalde gedragingen en gebeurtenissen niet goed kunnen herinneren, wat tot incorrecte rapportage leidt. Ook kan het voorkomen dat bepaalde groepen mensen zich gedragingen en gebeurtenissen verschillend herinneren, bijvoorbeeld wanneer mensen die voedselonzekerheid ervaren meer gefocust zijn op voeding en zich daardoor hun voedinginname anders herinneren en rapporteren dan mensen die geen voedselonzekerheid ervaren.

## Conclusie

De COVID-19-crisis hangt samen met veranderingen in de ervaren voedselzekerheid in een Nederlandse populatie met een relatief lage sociaaleconomische positie. Een deel van de deelnemers ervaarde sinds het begin van de COVID-19-crisis een lagere voedselzekerheid, maar er waren ook personen die juist een hogere mate van voedselzekerheid ervaren, wat mogelijk verklaard werd door terugval op een sterk sociaal netwerk. Personen met een verminderde ervaren voedselzekerheid voldeden over het algemeen minder goed aan de richtlijnen voor een gezond eetpatroon. Ook rapporteerden juist personen met een verminderde ervaren voedselzekerheid relatief vaker veranderingen in hun eetgedrag sinds het begin van de COVID-19-crisis, zoals een minder gezond eetpatroon en kleinere groente- en fruitconsumptie. Een goedkoper voedselaanbod van gezonde producten werd als een veelbelovende oplossing gezien, terwijl voedselbankgebruik weinig werd genoemd als mogelijke hulp voor een gezonder eetpatroon. Omdat de COVID-19-crisis leidt tot financiële onzekerheid bij veel mensen en ook tot veranderingen in de beschikbaarheid van voedsel, bekende risicofactoren voor een verminderde voedselzekerheid, benadrukken deze bevindingen hoe belangrijk het is om juist nu maatregelen te nemen om de voedselzekerheid te verhogen (waarbij ook gedacht moet worden aan het verbeteren of versterken van sociale steun en sociale cohesie), en passende hulp en ondersteuning te bieden bij een gezond eetpatroon, vooral aan mensen die een verminderde voedselzekerheid ervaren. Het is dan ook essentieel om met passende oplossingen te komen, die aansluiten bij de behoeften van personen met een verminderde ervaren voedselzekerheid, om zodoende de schadelijke langetermijngevolgen van een verminderde voedselzekerheid voor de gezondheid tegen te gaan.
