# Supplementary material for: Changes in perceived food insecurity and eating behavior in the Netherlands since the COVID-19-crisis
Source: TSG. 2021 Jul 23;99(3):83–94. [Article in Dutch] doi: 10.1007/s12508-021-00311-0 (PMC8299741; doi:10.1007/s12508-021-00311-0)
Supplement: Supplementary file 1 [file 12508_2021_311_MOESM1_ESM.pdf]

Digitaal aanvullende content behorende bij het artikel *Veranderingen in ervaren voedselzekerheid en eetgedrag in Nederland sinds de COVID-19-uitbraak*

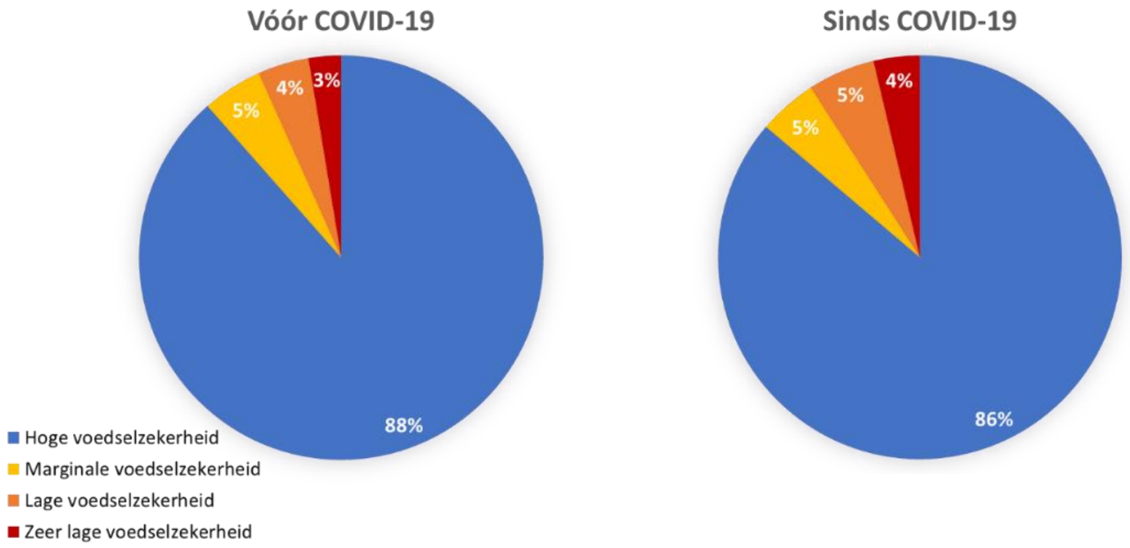

Aanvullende Figuur 1. Status van voedselzekerheid vóór en sinds de COVID-19 crisis

Aanvullende tabel 1. Vragenlijst over voedselzekerheid die gebruikt is in het huidige onderzoek (uitgestuurd in december 2020)

Voedselzekerheid op dit moment (sinds de Corona uitbraak)

De volgende stellingen gaan over de voedselinname in uw huishouden en of u het eten dat u nodig had, kon betalen. De eerste stellingen gaan over de periode **sinds de Corona uitbraak** (vanaf maart dit jaar).

Geef aan hoe vaak onderstaande stellingen op u van toepassing zijn.

1 **In de periode sinds de Corona uitbraak was het eten dat ik (of andere gezinsleden) kocht(en) niet genoeg, en ik (of andere gezinsleden) had(den) geen geld om meer eten te kopen.**

- ○ dit kwam vaak voor
- ○ dit kwam soms voor
- ○ dit kwam nooit voor
- ○ weet ik niet / wil ik niet zeggen

2 **In de periode sinds de Corona uitbraak had ik (of andere gezinsleden) niet genoeg geld om een gezonde maaltijd te eten.**

- ○ dit kwam vaak voor
- ○ dit kwam soms voor
- ○ dit kwam nooit voor
- ○ weet ik niet / wil ik niet zeggen

3 **In de periode sinds de Corona uitbraak heb ik (of andere gezinsleden) wel eens minder gegeten of een maaltijd overgeslagen omdat er niet genoeg geld voor eten was.**

- ○ ja
- ○ nee **(→ ga door naar vraag 4)**
- ○ weet ik niet / wil ik niet zeggen **(→ ga door naar vraag 4)**

3a. **Hoe vaak kwam dit in de afgelopen maanden (sinds de Corona uitbraak) voor?**

- ○ (bijna) elke maand
- ○ sommige maanden maar niet elke maand
- ○ 1 of 2 maanden
- ○ weet ik niet / wil ik niet zeggen

4. **In de periode sinds de Corona uitbraak heb ik (of andere gezinsleden) wel eens te weinig gegeten omdat er niet genoeg geld voor eten was.**

- ○ ja
- ○ nee
- ○ weet ik niet / wil ik niet zeggen

5. **In de periode sinds de Corona uitbraak heb ik (of andere gezinsleden) wel eens honger gehad, maar niet gegeten omdat ik (of andere gezinsleden) niet meer eten kon(den) kopen.**

- ○ ja
- ○ nee
- ○ weet ik niet / wil ik niet zeggen

Voedselzekerheid vóór de Corona uitbraak

Nu volgen dezelfde stellingen als die u net heeft beantwoord over voedselzekerheid, maar dan gedurende **de 12 maanden vóór de Corona uitbraak** (de periode tussen maart 2019 en maart 2020).

Geef aan hoe vaak onderstaande stellingen op u van toepassing zijn.

1 **In de 12 maanden vóór de Corona uitbraak was het eten dat ik (of andere gezinsleden) kocht(en) niet genoeg, en ik (of andere gezinsleden) had(den) geen geld om meer eten te kopen.**

- ○ dit kwam vaak voor
- ○ dit kwam soms voor
- ○ dit kwam nooit voor
- ○ weet ik niet / wil ik niet zeggen

2 **In de 12 maanden vóór de Corona uitbraak had ik (of andere gezinsleden) niet genoeg geld om een <u>gezonde</u> maaltijd te eten.**

- ○ dit kwam vaak voor
- ○ dit kwam soms voor
- ○ dit kwam nooit voor
- ○ weet ik niet / wil ik niet zeggen

3 **In de 12 maanden vóór de Corona uitbraak heb ik (of andere gezinsleden) wel eens minder gegeten of een maaltijd overgeslagen omdat er niet genoeg geld voor eten was.**

- ○ ja
- ○ nee **(→ ga door naar vraag 4)**
- ○ weet ik niet / wil ik niet zeggen **(→ ga door naar vraag 4)**

3a. **Hoe vaak kwam dit in de 12 maanden vóór de Corona uitbraak voor?**

- ○ (bijna) elke maand
- ○ sommige maanden maar niet elke maand
- ○ 1 of 2 maanden
- ○ weet ik niet / wil ik niet zeggen

4 **In de 12 maanden vóór de Corona uitbraak heb ik (of andere gezinsleden) wel eens te weinig gegeten omdat er niet genoeg geld voor eten was.**

- ○ ja
- ○ nee
- ○ weet ik niet / wil ik niet zeggen

5 **In de 12 maanden vóór de Corona uitbraak heb ik (of andere gezinsleden) wel eens honger gehad, maar niet gegeten omdat ik (of andere gezinsleden) niet meer eten kon(den) kopen.**

- ○ ja
- ○ nee
- ○ weet ik niet / wil ik niet zeggen

Aanvullende tabel 2. Voedingscomponenten met bijbehorende voedingsrichtlijnen, bijdragepercentages en puntenwaardering per component

**Voedings-component**	**Voedingsrichtlijnen van de Nederlandse Gezondheidsraad en/of het Voedingscentrum**	**% bijdrage aan de voedings-component score**	**Eenheden**	**Voedingscomponent score**		
				**0 punten**	**5 punten**	**10 punten**
**Groenten**	Eet dagelijks ten minste 200 gram groente	100	Gram/d	0	*Continue*	≥200
**Fruit**	Eet dagelijks ten minste 200 gram (2 porties) fruit	100	Portie/ d	0	*Continue*	≥ 2
**Peulvruchten**	Eet wekelijks een portie peulvruchten	100	Gram/w	0	*Continue*	≥ 135
**Ongezouten noten**	Eet ten minste 15 gram ongezouten noten per dag	100	Gram/d	0	*Continue*	≥ 15
**Vis**	Eet een keer per week vis	50	Portie/w	0	<1	≥ 1
	Bij voorkeur vette vis	50	-	Geen vis	Magere of zowel vette als magere vis	Vooral vette vis
**Graan-producten**	Eet dagelijks ten minste 90 gram volkorenproducten	50	Gram/d	0	*Continue*	≥ 90
	Vervang geraffineerde graanproducten door volkorenproducten	50	-	Vooral geraffineerde graanproducten	Zowel geraffineerde als volkoren graanproducten	Vooral volkoren graanproducten
**Zuivel**	Eet dagelijks 2-3 porties zuivel	50	Portie/d	0	*Continue*	≥ 2
	Bij voorkeur halfvolle/ magere zuivel	50	-	Vooral vette zuivel	Zowel vette als halfvolle/ magere zuivel	Vooral halfvolle/ magere zuivel
**Thee**	Drink dagelijks 3-4 koppen groene/ zwarte thee	100	Portie/d	<1 EN vooral groene/ zwarte thee;<br><br><3 EN zowel kruidenthee als groene/ zwarte thee;<br><br>Geen thee gedronken;<br><br>Vooral kruidenthee	1-2 EN vooral groene/ zwarte thee;<br><br>≥ 3 EN zowel kruidenthee als groene/ zwarte thee	≥ 3 EN vooral groene/ zwarte thee

**Koffie**	Vervang ongefilterde door gefilterde koffie	100	-	Kookkoffie/ Turkse of Griekse koffie; koffie gezet met een cafetière;	Koffie uit automaat*; koffie van cups; espresso	Geen koffie gedronken of filterkoffie, koffie van pads, oploskoffie
**Oliën en vetten**	Vervang boter, harde margarine en bak- en braadvetten door zachte margarine, vloeibaar bak- en braadvet en plantaardige oliën	50		Boter, harde margarine		Vloeibaar bak- en braadvet, plantaardige oliën
		50	-	Boter op brood of brood wordt niet besmeerd	Halfvolle boter of harde margarine op brood	Zachte margarine op brood
**Suiker-houdende dranken**	Drink zo min mogelijk suikerhoudende dranken	100	Portie/d	≥ 1	<1	0
**Hartige snacks**	Voor producten buiten de Schijf van vijf: kies niet vaker dan 3-5 keer per dag iets uit de dagkeuzes, en niet vaker dan 3 keer per week iets uit de weekkeuzes.	50	Grote portie/w	≥ 3	<1 to 2	0
		50	Kleine portie/d	> 3	*Continue*	0
**Zoete snacks**	Voor producten buiten de Schijf van vijf: kies niet vaker dan 3-5 keer per dag iets uit de dagkeuzes, en niet vaker dan 3 keer per week iets uit de weekkeuzes.	100	Portie/w	≥ 3	<1 to 2	0

Afkortingen: d=dag; w=week; *Koffie uit een automaat kan zowel gefilterde als ongefilterde koffie zijn. Aangezien het in de koffieautomaat gebruikte filter niet bekend is, wordt aangenomen dat het cafestolgehalte matig is.

Aanvullende tabel 3. Bevestigende antwoorden op de stellingen betreffende voedselzekerheid, vóór en sinds de COVID-19 crisis

**Stellingen over ervaren voedselzekerheid**	**Vóór COVID-19 crisis**	**Sinds COVID-19 crisis**
	n (%) bevestigend geantwoord	n (%) bevestigend geantwoord
Het eten dat ik (of andere gezinsleden) kocht(en) was niet genoeg, en ik (of andere gezinsleden) had(den) geen geld om meer eten te kopen.	67 (6,5)	94 (9,1)
Ik (of andere gezinsleden) had(den) niet genoeg geld om een gezonde maaltijd te eten.	106 (10,3)	125 (12,1)
Ik (of andere gezinsleden) heb(ben) wel eens minder gegeten of een maaltijd overgeslagen omdat er niet genoeg geld voor eten was.	39 (3,8)	53 (5,1)
Hoe vaak kwam dit voor: sommige maanden of vaker?	34 (87,2) van de 39*	43 (81,1) van de 53*
Ik (of andere gezinsleden) heb(ben) wel eens te weinig gegeten omdat er niet genoeg geld voor eten was.	47 (4,5)	63 (6,1)
Ik (of andere gezinsleden) heb(ben) wel eens honger gehad, maar niet gegeten omdat ik (of andere gezinsleden) niet meer eten kon(den) kopen.	26 (2,5)	31 (3,0)

*Het aantal en percentage deelnemers dat aangeeft dat zij sommige maanden of vaker “minder gegeten hebben of een maaltijd overgeslagen omdat er niet genoeg geld voor eten was”
